# Editorial: Food Melanoidins: Chemistry and Nutrition

**DOI:** 10.3389/fnut.2022.881690

**Published:** 2022-03-25

**Authors:** Fernando M. Nunes, Maria Dolores Del Castillo, Franck Carbonero

**Affiliations:** ^1^Chemistry Research Center—Vila Real, Food and Wine Chemistry Lab, University of Trás-os-Montes e Alto Douro, Vila Real, Portugal; ^2^Spanish National Research Council, Madrid, Spain; ^3^Department of Nutrition and Exercise Physiology, Elson Floyd College of Medicine and School of Food Science, Washington State University, Spokane, WA, United States

**Keywords:** food chemistry, food processing, function, Maillard reaction, melanoidins, bioactivity, nutrition, thermal processing

The thermal processing of food is as old as the discovery of fire by humankind. Amongst the many chemical reactions and physical transformations that occur in foods during their thermal processing, Maillard reaction has important implications in food quality, safety, and health effects. The currently accepted definition of melanoidins is the brown colored nitrogen-containing high molecular weight material formed as end products of the Maillard reaction. The brown color development of foods as a result of their thermal processing is largely due to the formation of melanoidins. Despite their abundance and importance in the diet, melanoidins are still the most enigmatic food macromolecule today, as their chemical structure is still largely unknown, being highly dependent of the food chemical composition (simple sugars/polysaccharides, protein/peptides/amino acids, phenolic compounds, etc.) (Figure 1).

**Figure 1:**
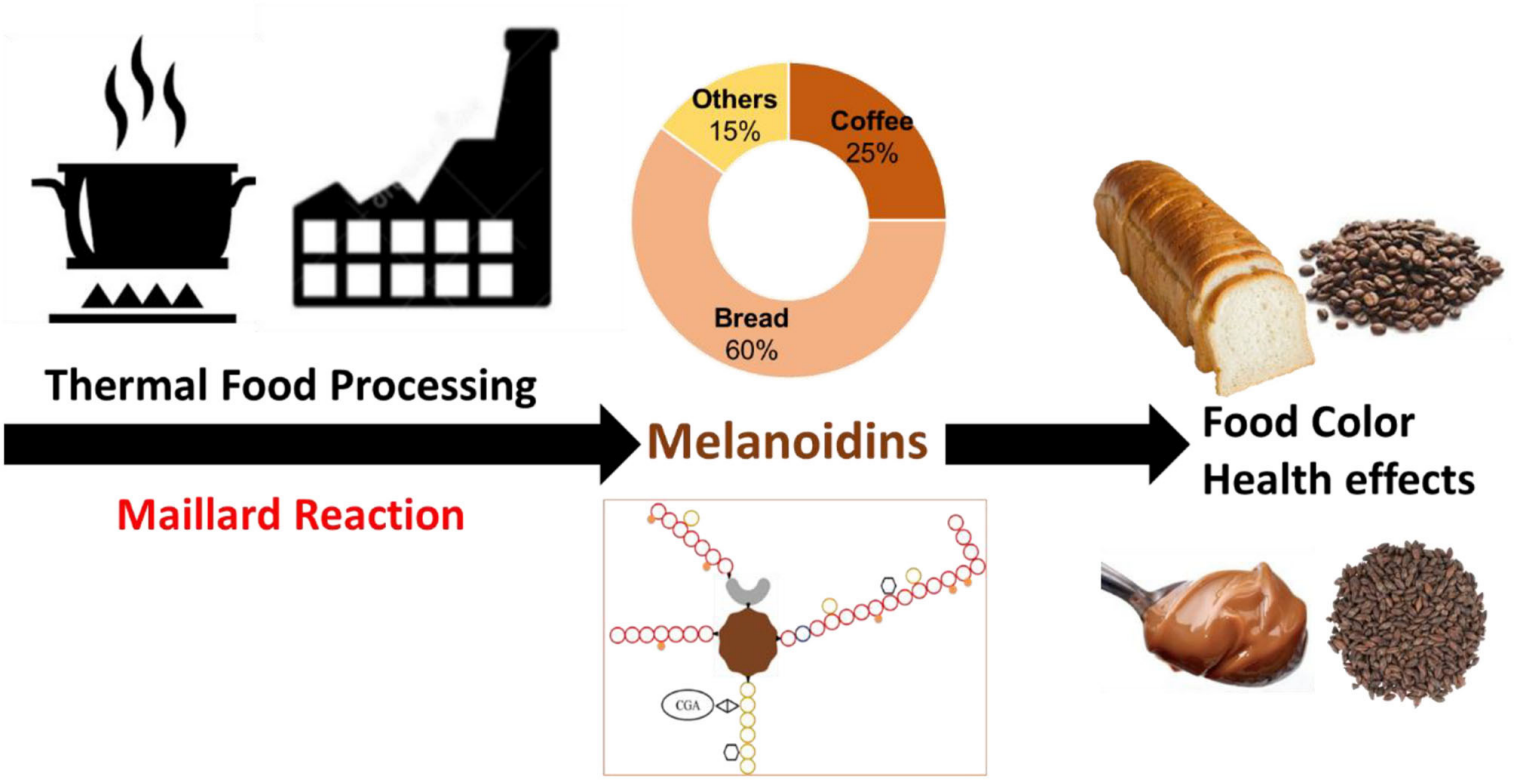
Nature, main dietary sources and relative contribution to melanoidin consumption, and functions of dietary melanoidins.

In the 21st century, 107 years after the first description of the Maillard reaction by the French chemist Louis Camille Maillard, there are still no clear answers to the following questions: what is the exact chemical structure of food melanoidins, and what is the mechanism of their formation? Different beneficial biological properties have been described for these compounds either from different foods or simplified in model systems. However, the lack of knowledge on their structure and mechanism of formation limits our understanding on their impact in nutrition and health. As a consequence, the interest on this field has greatly increased. The present Research Topic aims to contribute to this fundamental knowledge.

Melanoidins are widely distributed in our foods as part of our daily diet. They are present in coffee, baked goods, chips, cocoa, roasted barley, fortified wine, beer, grilled meat, and among others. Bakery products and coffee are the two primary sources of melanoidins in our diet providing about 6.0 and 1.5 g/day, respectively. Melanoidins have been also reported in various coffee by-products such as cascara, silverskin and spent coffee grounds (Iriondo-DeHond et al.) (Figure 1).

Food processing conditions can significantly affect the amount and structure of melanoidins present in the final product. Rodríguez et al. studied the effect of pH on the formation of melanoidins during dulce de leche production. Melanoidins with higher average MW after the enzymatic hydrolysis and darker colors were observed in the products produced at higher pH. A heterogeneous and complex composition of the melanoidins was observed even though structurally related. Results from 1H NMR showed a higher degree of aromaticity at higher pH values.

Several biological properties have been described for the melanoidins generated during coffee processing such as antioxidant, anti-inflammatory, dietary fiber effect, and prebiotic capacity, which make them very interesting from a nutritional point of view. The article of Iriondo-DeHond et al., a descriptive narrative mini-review, summarizes the nature, structure, digestibility, properties (sensory, nutritional, and health-promoting), safety and regulatory status of melanoidins from the coffee brew and its by-products.

Barley malt is the primary cereal malt used in beer production, a widely consumed beverage worldwide. Melanoidins are produced at different stages during the brewing process, enhancing the beers' flavor, texture, and sensory properties, making beer an important source of dietary melanoidins. The potential biological properties of barley melanoidins was reviewed by Sharma et al. Low digestibility of melanoidins leads to less availability to the organisms but is considered to function as dietary fiber that can be metabolized by the lower gut microbiota promoting the growth of Lactobacilli and Bifidobacteria in the gastrointestinal tract, preventing the colonization of potential pathogens.

The presence of bound phenolic compounds to melanoidins has been linked to many of their potential biological activities, for example, their reducing and antioxidant capacity. The work of Antonietti et al. investigated the presence of different types of bound phenolic compounds, ester-linked or condensed phenolic structures in instant soluble coffee and instant soluble barley. Melanoidins from instant soluble coffee presented a significantly higher content of condensed phenolic compounds, more than double compared to instant soluble barley, also showing a higher *in vitro* scavenging of ABTS·+ and NO radicals in line with their bound phenolic content. Nevertheless, instant soluble barley melanoidins presented, on average, a higher inhibitory effect on NO production from LPS-stimulated macrophages.

Alves et al. investigated the *in vitro* bioaccessibility and gut metabolism of free and melanoidin-bound phenolic compounds from coffee and bread. After the gastric and intestinal steps, the bioaccessibilities of all phenolics were, on average, 11 and 26%, for coffee and bread melanoidins, respectively. Bioaccessibilities of melanoidin-bound phenolics reached maximum values after gut fermentation for 24 h (50% for coffee and 51% for bread).

We hope this Research Topic, containing five quality articles, three research articles and two reviews, will further promote the interest in the chemistry and nutrition of food melanoidins.

## Author Contributions

All authors listed have made a substantial, direct, and intellectual contribution to the work and approved it for publication.

## Conflict of Interest

The authors declare that the research was conducted in the absence of any commercial or financial relationships that could be construed as a potential conflict of interest.

## Publisher's Note

All claims expressed in this article are solely those of the authors and do not necessarily represent those of their affiliated organizations, or those of the publisher, the editors and the reviewers. Any product that may be evaluated in this article, or claim that may be made by its manufacturer, is not guaranteed or endorsed by the publisher.

